# Antibiotic Exposure and Periodontal Susceptibility: A Risk-Modifying Hypothesis

**DOI:** 10.3390/ijms27125150

**Published:** 2026-06-06

**Authors:** Nada Tawfig Hashim, Nallan C. S. K. Chaitanya, Rasha Babiker, Ayman Ahmed, Muhammed Mustahsen Rahman, Riham Mohammed, Vivek Padmanabhan, Md Sofiqul Islam, Mariam Elsheikh, Salma Musa Adam Abduljalil, Bakri Gobara Gismalla, Shadi El Bahra

**Affiliations:** 1 Department of Periodontics, RAK College of Dental Sciences, RAK Medical & Health Sciences University, Ras-AlKhaimah 12973, United Arab Emirates; 2 Department of Oral Rehabilitation, Faculty of Dentistry, University of Khartoum, Khartoum 11115, Sudan; 3 Department of Oral Medicine and Radiology, RAK College of Dental Sciences, RAK Medical & Health Sciences University, Ras-AlKhaimah 12973, United Arab Emirates; 4 Department of Physiology, RAK College of Medical Sciences, RAK Medical and Health Sciences University, Ras-AlKhaimah 11172, United Arab Emirates; 5 Department of Periodontology and Implantology, Nile University, Khartoum 11115, Sudan; 6 Department of Oral Surgery, RAK College of Dental Sciences, RAK Medical & Health Sciences University, Ras-AlKhaimah 12973, United Arab Emirates; 7 Department of Pediatric and Preventive Dentistry, RAK College of Dental Sciences, RAK Medical & Health Sciences University, Ras-AlKhaimah 12973, United Arab Emirates; 8 Department of Operative Dentistry, RAK College of Dental Sciences, RAK Medical and Health Sciences University, Ras-AlKhaimah 12973, United Arab Emirates; sofiqul.islam@rakmhsu.ac.ae; 9 Department of Oral &Maxillofacial Surgery, Faculty of Dentistry, University of Khartoum, Khartoum 11115, Sudan; 10 Department of Periodontology, Faculty of Dentistry, National University, Khartoum 11115, Sudan; 11 Clinical Sciences Department, College of Dentistry, Ajman University, Ajman P.O. Box 346, United Arab Emirates

**Keywords:** antibiotics, periodontitis, dysbiosis, oral microbiome, gut–oral axis, immune regulation, bone remodeling, microbial homeostasis, host–microbe interactions, systemic dysbiosis

## Abstract

Systemic antibiotics are among the most widely prescribed therapeutic agents worldwide, and their effects on host–microbe equilibrium extend well beyond the infection for which they are intended. Periodontitis is conventionally framed as a biofilm-initiated, host-mediated inflammatory disease, although recent work has shifted this framework toward microbial homeostasis as a regulator of periodontal stability. We hypothesize that antibiotics are not direct etiologic agents of periodontitis but instead act as risk-modifying factors that lower the threshold at which plaque-mediated inflammation progresses to destructive disease. We propose that this effect may operate through several mechanisms: broad-spectrum or repeated exposure could deplete protective commensals and narrow microbial diversity, creating ecological space for opportunistic and pathogenic taxa; antibiotics may also alter host neutrophil function, cytokine profiles, and antimicrobial peptide regulation and may interfere with the osteoblastic and osteoclastic dynamics governing alveolar bone remodelling; and antibiotic-induced gut dysbiosis may propagate systemic inflammatory signals that further modulate periodontal susceptibility. To evaluate this hypothesis, we synthesize the available clinical, epidemiological, and experimental data across four converging axes—oral microbial ecology, immune regulation, alveolar bone remodelling, and the gut–oral axis—and identify the predictions the hypothesis generates and the evidence gaps it exposes. We emphasize that no clinical study has yet demonstrated a causal link between antibiotic exposure and periodontitis; the framework advanced here is therefore intended to inform antimicrobial stewardship in dentistry and to define a research agenda for determining whether antibiotic exposure constitutes a clinically meaningful modifier of periodontal disease susceptibility.

## 1. Introduction

**Figure 1:**
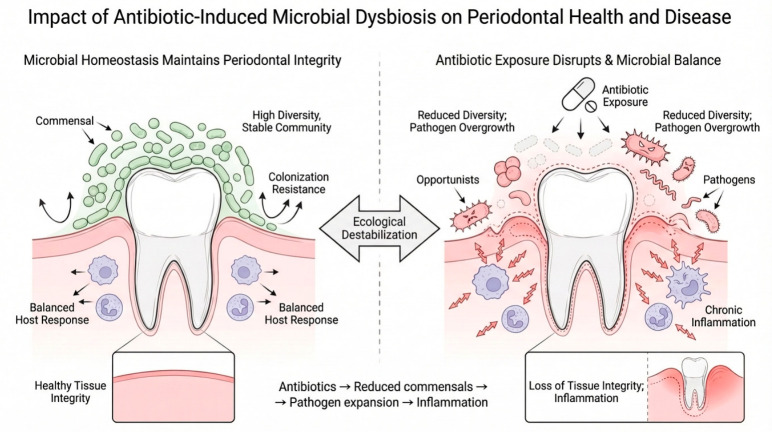
Illustrates the role of microbial homeostasis in maintaining periodontal integrity and the consequences of antibiotic-induced dysbiosis. Under physiologic conditions, a diverse and balanced commensal microbiota promotes colonization resistance and supports host immune equilibrium, preserving tissue integrity. Commensal bacteria contribute to ecological stability and prevent pathogenic overgrowth through competitive and immunomodulatory mechanisms. Antibiotic exposure disrupts this balance by reducing microbial diversity and eliminating protective commensals, thereby facilitating opportunistic pathogen expansion. This ecological destabilization triggers host inflammatory responses, leading to periodontal tissue damage, loss of structural integrity, and progression of periodontal disease. Figure created with FigureLab.

Repeated or prolonged exposure to systemic antibiotics is now recognized as a factor capable of reshaping both the oral and gut microbiomes, with consequences that extend to periodontal health [1,2]. Periodontitis is conventionally described as a biofilm-initiated, host-mediated inflammatory disease, but that paradigm has been broadened by work emphasizing microbial homeostasis as the central regulator of periodontal tissue integrity [3] (Figure 1). Broad-spectrum or prolonged antibiotic exposure can destabilize this equilibrium—reducing protective commensals, narrowing microbial diversity, and opening niches for opportunistic and pathogenic organisms [4]. The downstream consequences are weakened colonization resistance, reduced microbial resilience, and a biofilm shifted toward configurations that sustain chronic inflammation [5].

The effects of antibiotics on periodontal stability are not confined to the microbiota. Several antibiotic classes also engage host immunological and cellular processes that regulate periodontal homeostasis [6,7]. In experimental work, specific antibiotic classes modulate neutrophil activity, alter cytokine signalling, and disturb antimicrobial peptide balance, in turn changing the host’s capacity to regulate inflammatory responses to dental plaque [8]. Some antibiotics also interfere with osteoblast and osteoclast function, modify bone-cell viability, and disturb remodelling dynamics—indirect routes by which antibiotic exposure may shape alveolar bone responsiveness during inflammatory challenge [2]. Together, these microbial, immune, and tissue-level effects supply a biologically coherent route by which antibiotics may modify periodontal susceptibility [6,7,8].

Antibiotics are not direct etiologic agents of periodontitis. However, the clinical, epidemiological, and experimental literature converges on a more nuanced position: antibiotic-associated disruption of microbial and host homeostasis can heighten periodontal vulnerability in predisposed individuals [9]. Three lines of observation are consistent with this position: altered recolonization patterns after antibiotic exposure, raised inflammatory indices in patients receiving repeated antibiotic courses, and experimentally induced periodontal breakdown in animal models [10,11]. The pathways involved appear connected rather than parallel, forming a continuum in which antibiotic-induced microbial imbalance triggers immune dysregulation, metabolic disruption, and reduced tissue resilience downstream.

Rising global antibiotic consumption, accelerating antimicrobial resistance, and documented declines in microbial diversity push these questions beyond the level of the individual patient and into the public health domain [12]. The response sits at the level of antimicrobial stewardship—targeted prescribing in both dental and medical settings and preventive strategies aimed at preserving microbial homeostasis. Candidate approaches include indication-driven antibiotic use, microbiome-supportive adjuncts such as probiotics or postbiotics, and early identification of patients particularly vulnerable to antibiotic-induced microbial and immune shifts [13,14]. A clearer mechanistic picture should inform global guidelines that balance therapeutic efficacy against preservation of oral and systemic microbial ecosystems.

The evidence to date remains fragmented, and no single account has yet integrated the findings from microbiology, immunology, systemic dysbiosis research, and bone biology into a coherent mechanistic position. Here we advance the following hypothesis: antibiotic exposure does not initiate periodontitis directly but is hypothesized to function as a risk-modifying factor that may lower the threshold at which plaque-mediated inflammation progresses to destructive disease. We examine four converging axes through which this effect may operate—oral microbial ecology, immune regulation, alveolar bone remodelling, and the gut–oral axis—and we identify the predictions the hypothesis generates, the evidence gaps it exposes, and the testable research questions it implies. The objective is to determine whether antibiotic use is a meaningful modifier of periodontal susceptibility and to define the empirical work required to answer that question.

To make the hypothesis falsifiable, we translate it into the following specific, testable predictions. Because no direct clinical data yet exist, the effect sizes below are stated as quantitative thresholds against which the hypothesis can be confirmed or refuted, rather than as established values:

- Oral ecology. Repeated broad-spectrum β-lactam exposure (≥2 courses per year) will reduce subgingival commensal Streptococcus–Veillonella relative abundance by ≥30% and increase Porphyromonas gingivalis load ≥2-fold within 4–8 weeks of exposure, as measured by 16S/shotgun metagenomic sequencing.
- Clinical periodontal outcome. Adults with high cumulative antibiotic burden (≥5 courses over 5 years) will show ≥0.5 mm greater mean probing pocket depth and ≥0.5 mm greater clinical attachment loss than matched low-exposure controls, after adjustment for plaque score, smoking, and diabetes.
- Rebound colonization. During the 4–12-week post-antibiotic recolonization window, fluoroquinolone exposure will increase the prevalence of hypervirulent, SOS-response–induced strains and raise bleeding-on-probing by ≥15 percentage points relative to pre-treatment baseline.
- Immune regulation. Macrolide exposure will lower gingival crevicular fluid LL-37 and human β-defensin-2 concentrations by ≥25% and shift the local cytokine profile toward a Th17 signature (≥1.5-fold rise in IL-17 and IL-23) within 2 weeks.
- Alveolar bone remodelling. In experimental periodontitis (e.g., ligature) models, fluoroquinolone or aminoglycoside co-exposure will raise the RANKL/OPG ratio by ≥50% and increase alveolar bone loss by ≥20% (micro-CT) versus ligature-only controls, while producing no measurable bone loss in non-ligated animals.
- Gut–oral axis. Antibiotic-induced gut dysbiosis will reduce faecal short-chain fatty acids (butyrate) by ≥30% and raise circulating LPS and IL-6 by ≥25%, with these systemic markers correlating (r ≥ 0.4) with increased periodontal inflammatory indices.

A dose- and class-dependent gradient across these endpoints, together with partial reversibility following microbiome-restorative intervention, would support the hypothesis; the absence of such associations after adequate exposure and follow-up would refute it.

## 2. Literature Search Strategy and Evidence Appraisal

This article is a hypothesis-driven conceptual review and does not constitute a systematic review or meta-analysis. To assemble the evidence base, a structured narrative literature search was performed across PubMed/MEDLINE, Scopus, and Web of Science for records published between January 1991 and October 2025. This time frame was chosen to encompass the full span of literature relevant to the antibiotic–microbiome–periodontal axis, the earliest included study dating from 2000 and the most recent from 2025.

Search terms combined controlled vocabulary and free-text keywords using Boolean operators, including (“antibiotics” OR “antimicrobial agents” OR antibiotic classes such as “β-lactams,” “macrolides,” “fluoroquinolones,” “tetracyclines,” “aminoglycosides”) AND (“periodontitis” OR “periodontal disease” OR “alveolar bone loss” OR “gingival inflammation”) AND (“oral microbiome” OR “dysbiosis” OR “microbial homeostasis” OR “gut–oral axis” OR “immune regulation” OR “osteoclast” OR “osteoblast” OR “short-chain fatty acids”). The reference lists of relevant articles were hand-searched to identify additional sources.

Articles were included if they were peer-reviewed original studies (clinical, animal, or in vitro), systematic or narrative reviews, or mechanistic studies, published in English, that addressed the relationship between antibiotic exposure and oral or gut microbial ecology, host immune regulation, bone-cell biology, or periodontal outcomes. Non-peer-reviewed material, conference abstracts without an accessible full text, non-English articles, and studies without relevance to the antibiotic–microbiome–periodontal axis were excluded.

The evidence considered spanned the full hierarchy—human clinical and epidemiological studies, animal models, in vitro and molecular experiments, and prior reviews—reflecting the breadth required to construct an integrative mechanistic hypothesis. 

## 3. Antibiotic-Induced Dysbiosis as a Pathway Toward Periodontal Disease

Broad-spectrum or repeated antibiotic therapy may disrupt the microbial balance of the oral cavity, potentially increasing susceptibility to periodontal inflammation [15]. The ecological vacancy that follows may favor recolonization by organisms associated with periodontal inflammation and tissue damage [15]. Antibiotics deplete pathogenic organisms but also remove keystone commensals such as *Streptococcus sanguinis*, *S. gordonii*, *Veillonella*, and *Actinomyces* species that maintain ecological stability through competitive exclusion, hydrogen peroxide production, alkali generation, and cooperative interactions with early colonizers [16]. Their loss narrows microbial diversity and dismantles the metabolic cross-feeding networks that normally restrain proteolytic and asaccharolytic anaerobes. In the resulting ecological vacancy, opportunistic taxa including *Porphyromonas gingivalis*, *Tannerella forsythia*, *Filifactor alocis*, and other Gram-negative anaerobes expand more readily, shifting the community toward a dysbiotic, inflammation-prone configuration enriched in virulence-associated traits [17,18].

**Figure 2:**
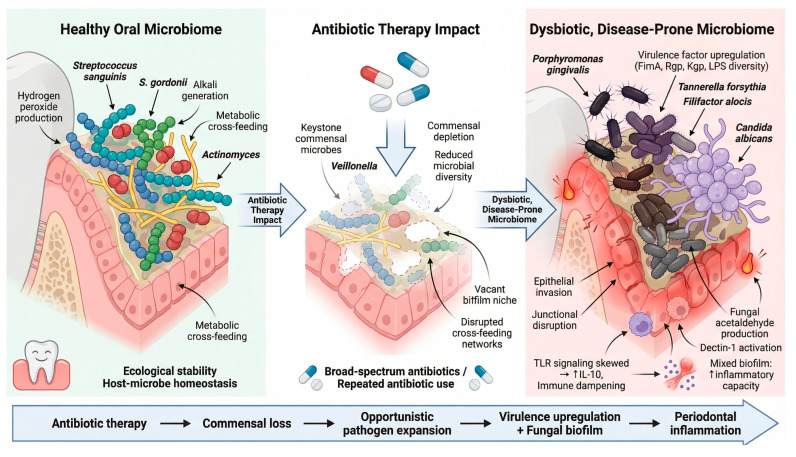
Depicts the transition from a healthy oral microbiome to antibiotic-induced dysbiosis and subsequent periodontal inflammation. In the healthy state, diverse commensal microorganisms engage in metabolic cross-feeding, hydrogen peroxide production, and ecological cooperation, maintaining host–microbe homeostasis and epithelial integrity. Antibiotic therapy disrupts this balance by depleting keystone commensals, reducing microbial diversity, and creating vacant niches that favor opportunistic colonization. This ecological shift promotes the expansion of virulent bacterial species such as Porphyromonas gingivalis and Tannerella forsythia, along with fungal overgrowth including Candida albicans. The resulting dysbiotic biofilm enhances virulence factor expression, impairs epithelial barrier function, and dysregulates immune signaling, ultimately driving periodontal inflammation and tissue destruction. Figure created with FigureLab.

At the molecular level, antibiotic-driven dysbiosis intensifies the pathogenic potential of the resident microbiota. *P. gingivalis* upregulates fimbrial adhesins (FimA), cysteine proteinases (Rgp/Kgp), and structurally diverse LPS isoforms that enhance epithelial invasion, disrupt junctional integrity, and skew Toll-like receptor signaling toward a dampened, IL-10–tolerant state that permits deeper microbial penetration [19,20]. Fungal expansion particularly *Candida albicans* amplifies dysbiosis by generating acetaldehyde, activating dectin-1 through β-glucan exposure, and forming mixed bacterial–fungal biofilms with superior structural resilience and increased inflammatory capacity [21] (Figure 2).

**Figure 3:**
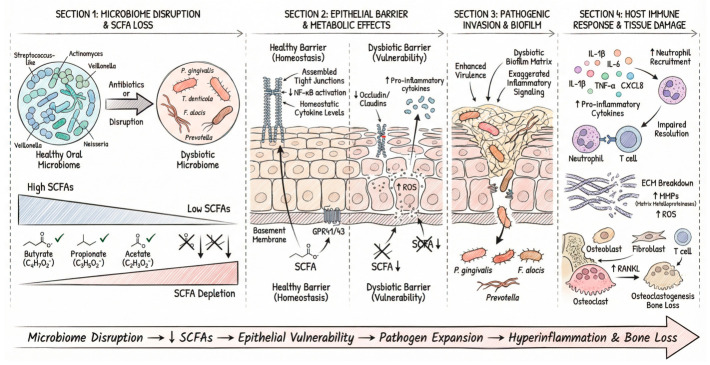
Illustrates the mechanistic cascade linking microbiome disruption to periodontal tissue destruction. Antibiotic-induced microbial imbalance reduces beneficial short-chain fatty acid (SCFA) production, impairing epithelial barrier integrity and weakening tight junction proteins such as claudins and occludin. This epithelial vulnerability facilitates pathogenic biofilm invasion and enhances virulence factor activity, promoting bacterial proliferation and immune evasion. The dysbiotic microbiome stimulates exaggerated host immune responses characterized by increased pro-inflammatory cytokine release, neutrophil infiltration, and activation of osteoclast-mediated bone resorption. Collectively, these interconnected processes drive hyperinflammation, connective tissue breakdown, and progressive alveolar bone loss characteristic of periodontitis. Figure created with FigureLab.

During the recolonization phase that follows antibiotic-induced microbiome disruption, the loss of dominant commensals reshapes the metabolic landscape of the oral niche. A key consequence is the decline in commensal-derived short-chain fatty acids (SCFAs) such as butyrate, propionate, and acetate remove trophic and immunoregulatory cues that maintain epithelial homeostasis [22]. These metabolites support tight-junction assembly, oxygen-gradient maintenance, and epithelial nutrient metabolism through GPR41/GPR43 signalling and HDAC inhibition. Their loss therefore removes regulation of epithelial barrier strength and NF-κB activation, sensitizing the tissues to inflammatory triggers [23]. In this destabilized state, epithelial cells show reduced expression of occludin and claudins, heightened reactive oxygen species generation, and exaggerated cytokine responses to low-level microbial stimulation, creating a permissive environment for opportunistic periodontal pathogens such as *P. gingivalis*, *T. denticola*, and *F. alocis* to expand, acquire stronger virulence phenotypes, and drive sustained inflammatory signalling that accelerates tissue breakdown. SCFA depletion is therefore an early molecular event that amplifies both epithelial vulnerability and dysbiosis-driven inflammatory reprogramming [24,25] (Figure 3). These ecological and molecular disturbances raise host inflammatory responsiveness [26]. Dysbiotic biofilms strongly stimulate gingival epithelial and immune cells, increasing production of IL-1β, IL-6, TNF-α, and CXCL8, which amplifies neutrophil recruitment yet impairs resolution due to the absence of commensal-derived immunoregulatory cues. This hyper-inflammatory environment enhances matrix metalloproteinase activation, reactive oxygen species generation, and extracellular matrix degradation. In parallel, dysbiotic consortiums promote RANKL expression by periodontal ligament fibroblasts, osteoblast-lineage cells, and T lymphocytes, accelerating osteoclastogenesis and alveolar bone resorption [27,28].

Antibiotic-induced dysbiosis therefore does not damage periodontal tissue directly. It acts through a multi-level cascade—ecological destabilization, enhanced microbial virulence, epithelial barrier compromise, and dysregulated host inflammatory and osteoclastogenic signalling [29]—that lowers the threshold for periodontal tissue destruction in susceptible patients.

## 4. Rebound Colonization and Potential Enrichment of More Virulent Strains

**Figure 4:**
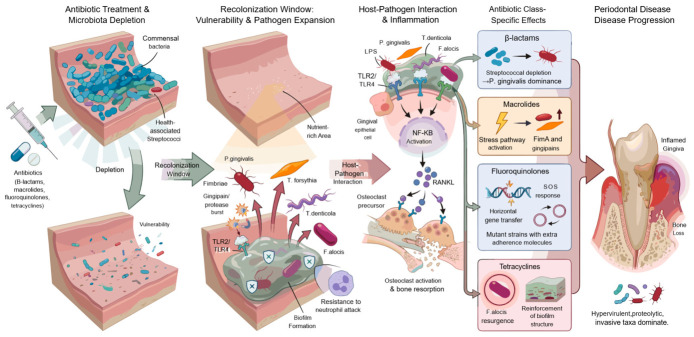
Illustrates the sequential impact of antibiotic therapy on oral microbial ecology and periodontal disease progression. Initial antibiotic exposure depletes commensal microbiota, disrupting ecological balance and creating a vulnerable recolonization window favoring opportunistic pathogens. Pathogenic bacteria and fungi expand during this period, activating host inflammatory pathways such as NF-κB and MAPK signaling, which increase pro-inflammatory cytokine production and tissue damage. Antibiotic class–specific effects further modulate microbial composition, influencing resistance development, virulence expression, and immune interactions. These combined microbial and host responses may accelerate periodontal disease progression, characterized by connective tissue degradation, pocket formation, and alveolar bone loss. Figure created with FigureLab.

The period that follows antibiotic-induced depletion of the oral microbiota may be biologically distinct. Evidence from in vitro and microbiological studies suggests that microbial succession during this recolonization phase could be reshaped toward organisms with stronger pathogenic potential [30]; this proposed rebound phenomenon has not yet been demonstrated clinically in the periodontal setting. The pathophysiology differs from the initial commensal-loss dysbiosis: in the rebound phase, key periodontal pathogens hold a selective ecological advantage as they reoccupy an unbalanced, nutrient-rich environment with reduced microbial competition [31]. In this context, species such as *P. gingivalis*, *T. forsythia*, *T. denticola*, and Filifactor alocis (*F. alocis*) may re-emerge with intensified virulence signatures, including upregulated fimbrial adhesins, increased gingipain and protease activity, and structural modifications in lipopolysaccharide that potentiate Toll-like receptor 2/4 engagement [32]. In experimental systems, these molecular adaptations have been reported to support faster and more cohesive biofilm maturation, with increased extracellular polymeric matrix production and greater resistance to neutrophil-mediated clearance, although the magnitude of these effects in vivo is unknown. In vitro, host cells exposed to post-antibiotic communities have shown heightened RANKL expression and amplified NF-κB activation, which could lower the inflammatory threshold for osteoclastogenesis and connective-tissue degradation [33]. The pattern and severity of any rebound virulence may vary by antibiotic class, although the supporting data are derived largely from in vitro and microbiological models. Broad-spectrum β-lactams disproportionately deplete health-associated streptococci while sparing several anaerobes, creating early recolonization niches in which *P. gingivalis* may gain ecological dominance [34]. Macrolides, despite their therapeutic utility in periodontics, can induce stress-response pathways in surviving *P. gingivalis* that upregulate FimA and gingipains, facilitating aggressive reattachment and enhanced biofilm cohesion [35]. Fluoroquinolones activate bacterial SOS responses, promoting horizontal gene transfer, mutational adaptation, and expansion of strains with stronger adherence and immune-evasive phenotypes [36]. Even tetracycline derivatives known for anti-collagenase effects may inadvertently facilitate the resurgence of *F. alocis*, a species with intrinsic resistance traits and a well-established role in the structural reinforcement of mature subgingival biofilms [37]. Taken together, these class-specific selective pressures are hypothesized to favor more virulent, proteolytic, and invasive taxa during recolonization. Whether the recolonization period itself constitutes a clinically meaningful driver of periodontal susceptibility and accelerated disease progression remains unproven and requires direct testing in human studies (Figure 4).

## 5. Antibiotics Alter Immune Homeostasis

The effects of antibiotics on periodontal tissues are not limited to their action on microbial communities. Several antibiotic classes also exert direct immunomodulatory actions that shift the periodontium toward a pro-inflammatory and bone-destructive state [38]. These actions converge on the innate immune network that maintains gingival equilibrium, particularly on neutrophil recruitment, chemotaxis, and reactive oxygen species regulation. β-lactams reduce neutrophil degranulation and opsonophagocytic killing, producing a functional neutropenia that weakens early containment of periodontal pathogens [39]. Macrolides, although classically considered anti-inflammatory, suppress Toll-like receptor signalling at high or prolonged exposures and lower the production of antimicrobial peptides such as human β-defensin-2 and LL-37 [40,41]. The result is weakened epithelial barrier defense and deeper microbial penetration into the sulcus [42]. Fluoroquinolones perturb immune homeostasis through a different route: by inducing mitochondrial oxidative stress in immune cells, raising NLRP3 inflammasome activation, and amplifying IL-1β and IL-18 release to low-grade microbial stimuli. The resulting cytokine signals drive exaggerated RANKL expression by periodontal ligament fibroblasts and gingival T cells, supporting osteoclastogenesis independent of microbial load [43,44].

**Figure 5:**
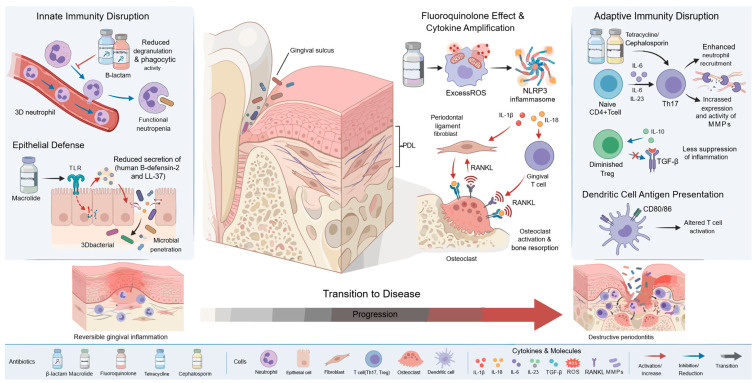
Illustrates the transition from periodontal health to disease following antibiotic-induced immune and epithelial disruption. Antibiotic exposure impairs innate immune functions, including neutrophil phagocytosis and antimicrobial peptide production, while weakening epithelial barrier integrity through reduced tight junction stability. Fluoroquinolone-mediated microbial and host alterations activate inflammatory pathways such as NLRP3 inflammasome signaling and increase pro-inflammatory cytokine release. Concurrent disruption of adaptive immunity leads to enhanced Th17 polarization, reduced regulatory T-cell function, and increased osteoclast activation. These coordinated immune dysregulations promote chronic inflammation, connective tissue degradation, and progressive alveolar bone loss characteristic of periodontitis. Figure created with FigureLab.

Adaptive immunity is affected in parallel. Recurrent courses of tetracyclines or cephalosporins have been linked to a Th17-dominant milieu driven by increased IL-6 and IL-23 signalling, which raises neutrophil recruitment and matrix metalloproteinase secretion at the gingival interface [45,46]. Regulatory T-cell (Treg) activity is reduced in parallel—partly through lower IL-10 production and impaired TGF-β receptor signalling—depriving periodontal tissues of the suppressive mechanisms that normally terminate inflammation [47]. Antibiotic-associated changes in dendritic cell antigen presentation extend the disturbance, altering costimulatory molecule expression (CD80/CD86) and promoting a more inflammatory T-cell phenotype. The combined innate and adaptive perturbations shift the gingival microenvironment toward disproportionate responsiveness to bacterial challenge, facilitating the transition from reversible gingival inflammation to destructive periodontal disease [6,48,49] (Figure 5).

## 6. Effects on Bone Cells and Alveolar Bone Remodeling

Antibiotics influence the structural dynamics of the periodontium by altering the physiology of bone-forming and bone-resorbing cells, changing the capacity of alveolar bone to withstand inflammatory insult [50]. Several antibiotic classes impair osteoblast proliferation, differentiation, and survival through distinct molecular routes. Fluoroquinolones induce mitochondrial dysfunction and reactive oxygen species generation in osteoblasts, activating intrinsic apoptotic cascades via cytochrome-c release and caspase-9 signalling [51]. The same oxidative burden suppresses RUNX2 and osteocalcin expression, directly compromising matrix synthesis and mineral deposition [52]. Aminoglycosides act differently: by binding ribosomal RNA and inhibiting protein translation, they reduce osteoblastic production of type I collagen and alkaline phosphatase, impairing mineralization and new bone formation [53]. At higher or prolonged exposures, they also disrupt the Wnt/β-catenin pathway, a central regulator of osteoblastic differentiation [54]. Certain tetracycline derivatives despite their recognized antimicrobial and matrix metalloproteinase-inhibitory benefits at low doses may inhibit osteoblast maturation when present at supratherapeutic levels by suppressing osterix expression and interfering with calcium-dependent signaling required for mineral apposition [55,56]. Some macrolides attenuate osteoclastic precursor fusion through disruption of NF-κB activation, altering the normal coupling between bone resorption and formation [57].

**Figure 6:**
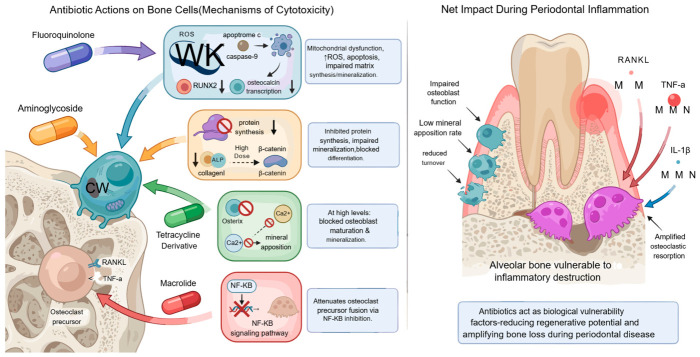
Illustrates the cytotoxic effects of antibiotics on bone cells and their net impact during periodontal inflammation. Antibiotic classes such as fluoroquinolones, aminoglycosides, tetracyclines, and macrolides influence osteoblast and osteoclast function through mechanisms including oxidative stress, mitochondrial dysfunction, altered signaling pathways, and impaired matrix synthesis. These effects disrupt bone remodeling by reducing osteoblast viability and enhancing osteoclast-mediated bone resorption. During periodontal inflammation, antibiotic-induced immune modulation further amplifies pro-inflammatory cytokine production, including IL-1β and RANKL, promoting osteoclast activation. Collectively, these mechanisms increase alveolar bone vulnerability and contribute to progressive bone loss in periodontal disease. Figure created with FigureLab.

These antibiotic-related changes do not initiate periodontitis on their own. They influence the trajectory of alveolar bone remodelling once inflammation is present. A compromised osteoblast population, reduced mineral apposition rate, or impaired turnover capacity makes alveolar bone more vulnerable to inflammatory osteoclastogenesis driven by RANKL, TNF-α, and IL-1β. Antibiotics in this setting act as biological vulnerability factors, diminishing the regenerative and reparative potential of bone tissues and amplifying the net effect of periodontal inflammation on bone resorption [58,59]. Microbial dysbiosis and immune dysregulation remain the primary drivers of periodontal breakdown, but antibiotic-induced changes in bone cell biology create a permissive environment in which the destructive phases of periodontitis progress more rapidly and with reduced capacity for spontaneous recovery (Figure 6).

## 7. Gut–Oral Axis: Systemic Dysbiosis → Periodontal Susceptibility

**Figure 7:**
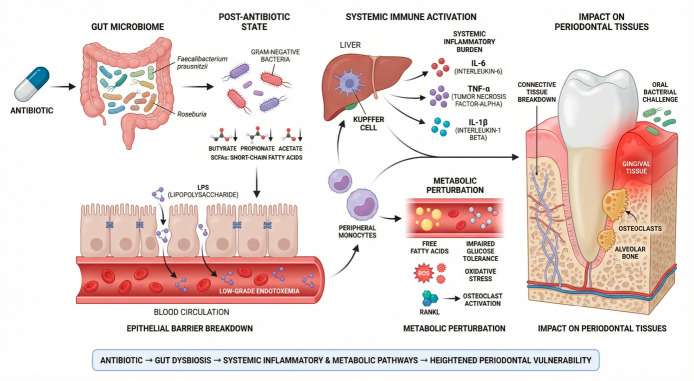
Illustrates the systemic consequences of antibiotic-induced gut microbiome dysbiosis and its impact on periodontal tissues. Antibiotic exposure disrupts gut microbial balance, reducing beneficial commensals and increasing circulating bacterial components such as lipopolysaccharides due to epithelial barrier breakdown. These microbial products activate systemic immune responses, including Kupffer cell stimulation and increased production of pro-inflammatory cytokines such as IL-1β, IL-6, and TNF-α. Systemic inflammation and metabolic perturbations enhance osteoclast activity, connective tissue degradation, and immune dysregulation within periodontal tissues. Collectively, gut dysbiosis contributes to heightened periodontal vulnerability, promoting inflammation, alveolar bone resorption, and progression of periodontal disease. Figure created with FigureLab.

Antibiotic-induced changes in the gut microbiome exert systemic effects that can heighten periodontal vulnerability through metabolic, immune, and inflammatory pathways separate from the local oral ecosystem [60]. Disruption of intestinal microbial communities reduces short-chain fatty acid-producing taxa such as Faecalibacterium prausnitzii and Roseburia [61]. As described for the oral niche (Section 2), the resulting decline in butyrate, propionate, and acetate weakens epithelial barrier integrity; in the gut, this specifically supports translocation of LPS into the circulation, producing low-grade endotoxemia [62]. Systemic LPS exposure activates hepatic Kupffer cells and peripheral monocytes, raising the same pro-inflammatory cytokines described above (Section 2). The cumulative effect is a higher systemic inflammatory burden that primes peripheral tissues—including the periodontal connective tissues—for exaggerated responses [63]. Gut dysbiosis also disrupts metabolic homeostasis, impairs glucose tolerance, and raises circulating free fatty acids, all of which potentiate oxidative stress and amplify RANKL-mediated osteoclastogenic signalling [64]. These systemic effects may lower the baseline inflammatory threshold of the periodontium. Gingival tissues become more reactive to modest bacterial challenge, and the microenvironment becomes more conducive to collagen degradation, connective-tissue breakdown, and bone resorption [65,66]. Antibiotics do not initiate periodontal disease directly. Their capacity to disrupt gut microbial equilibrium nevertheless creates a pro-inflammatory systemic context that raises the host’s susceptibility to periodontal destruction (Figure 7).

## 8. Clinical Contexts in Which Antibiotic-Related Periodontal Vulnerability Is Plausible but Not Yet Empirically Demonstrated

No clinical study to date has demonstrated a causal link between antibiotic exposure and the onset or progression of periodontitis. The associations discussed below are mechanistic and inferential and are presented as testable hypotheses, not as evidence of a proven clinical effect. Several lines of research nevertheless indicate that antibiotic exposure particularly when broad-spectrum, recurrent, or occurring during critical developmental windows may theoretically create ecological and immunological conditions that could increase susceptibility to periodontal inflammation [2,67,68]. These scenarios should be read as biologically plausible contexts in which the hypothesis can be tested, not as proven clinical outcomes.

Early-life antibiotic exposure consistently alters the pediatric oral and gut microbiome, reducing microbial diversity and delaying the maturation of commensal communities [69,70]. No studies have linked these disruptions directly to subsequent periodontitis, but several describe transient shifts in salivary microbial composition, and reduced colonization resistance after antibiotic courses [71,72,73]. These findings suggest that early perturbations may influence host–microbial interactions, although a causal relationship with future periodontal breakdown remains unproven.

In adults, the epidemiologic literature on repeated or long-term antibiotic use has focused primarily on systemic effects—gut dysbiosis, metabolic changes, and immune modulation [74,75]. A higher prevalence of oral candidiasis and shifts in salivary microbiota have been reported in chronically medicated populations [76], but no clinical study has yet demonstrated that antibiotic exposure independently causes periodontitis. Existing data suggest only that antibiotics may temporarily destabilize ecological equilibrium—particularly in patients with pre-existing risk factors such as smoking, diabetes, or poor oral hygiene [77].

Mechanistic insight comes principally from experimental models rather than human clinical studies. In rodents, broad-spectrum antibiotic regimens reduce microbial diversity, support overgrowth of opportunistic organisms, and raise inflammatory cytokine expression in gingival tissues. Some models show minor alveolar bone changes after microbiota depletion [78,79]. However, these effects typically occur in systems profoundly disrupted by high-dose antibiotics and may not reflect real-world clinical exposure.

The evidence is not uniformly consistent with a harmful effect, and a balanced appraisal must acknowledge findings that point in the opposite direction. In clinical practice, several antibiotic interventions improve periodontal outcomes rather than worsen them: adjunctive systemic amoxicillin–metronidazole combined with scaling and root planing produces greater reductions in probing pocket depth and gains in clinical attachment than mechanical therapy alone, and sub-antimicrobial-dose doxycycline exerts host-modulatory, matrix-metalloproteinase–inhibitory effects that benefit periodontal parameters. Other studies report only transient or negligible periodontal changes after antibiotic courses. These observations indicate that the relationship between antibiotic exposure and periodontal health is likely context-, dose-, spectrum-, and timing-dependent: the same agents that are therapeutic when targeted to an established infection may, under different conditions of broad-spectrum or repeated exposure, contribute to ecological disruption. This tension constrains the hypothesis and underscores that antibiotic exposure cannot be characterized as uniformly detrimental to the periodontium.

Taken together, the human and experimental data support a hypothesis in which antibiotic-driven ecological disruption raises susceptibility to periodontal inflammation under specific conditions. The hypothesis remains exactly that: no clinical study has yet demonstrated that antibiotic exposure independently causes periodontitis, nor that it acts as a stand-alone risk modifier.

Because the mechanistic claims advanced above draw on heterogeneous sources—and because much of the supporting work derives from in vitro systems or animal models using antibiotic concentrations that may exceed clinically achievable tissue levels. Table 1 grades the predominant evidence level underlying each key claim and notes its principal translational limitation. This grading is intended to make explicit which elements of the hypothesis rest on direct human data and which remain extrapolations requiring confirmation.

## 9. Future Directions

Table 2 summarizes the current evidence across the four mechanistic domains examined in this article, the predominant type of evidence supporting each, and the principal knowledge gaps that define the research agenda outlined below.

The evidence supporting the hypothesis advanced here is fragmented and largely inferential. Testing whether antibiotic exposure is a clinically meaningful modifier of periodontal susceptibility requires work along four lines. The first is longitudinal human cohort studies that delineate temporal relationships between antibiotic exposure patterns—dose, spectrum, timing, and cumulative burden—and subsequent changes in oral microbial ecology and periodontal outcomes. These studies should be paired with high-resolution metagenomic and metatranscriptomic analyses to characterise functional changes in recolonising microbiota and to identify pathogen-specific signatures associated with post-antibiotic hypervirulence.

The second is mechanistic investigation of how antibiotic-induced dysbiosis interacts with host immune and bone-remodelling pathways. Controlled in vivo models should quantify how each antibiotic class modulates neutrophil behavior, cytokine gradients, inflammasome activation, epithelial-barrier integrity, and RANKL–osteoclast signalling during early inflammatory challenge. Parallel work on the gut–oral axis is needed to determine how systemic consequences of intestinal dysbiosis—endotoxemia, metabolic perturbation, altered SCFA availability—translate into a heightened periodontal inflammatory tone. The distinction between local and systemic contributions to periodontal vulnerability depends on these data.

The third line is the identification of individual susceptibility phenotypes. Genetic polymorphisms affecting innate immunity, epithelial defense, or bone metabolism may amplify the periodontal consequences of antibiotic exposure, and host-genomic and epigenomic profiling should be integrated into future studies to identify at-risk subgroups. Pediatric and adolescent antibiotic exposure—coinciding with active immune, skeletal, and microbial development—deserves dedicated investigation, since early-life perturbation may produce long-lasting vulnerability to periodontal disease in adulthood.

The fourth line is translational. Microbiome-restorative strategies—targeted probiotics, synbiotics, microbial metabolite supplementation, and precision oral microbiome transplantation—should be evaluated for their capacity to mitigate the ecological and immunological shifts induced by antibiotics. Combined with refined antimicrobial stewardship policies, such interventions could reduce antibiotic-associated periodontal risk at the population level.

## 10. Conclusions

Antibiotics do not initiate periodontal disease in isolation. The hypothesis advanced in this article is that their capacity to disrupt microbial homeostasis, alter immune regulation, and impair bone-cell dynamics may create a biologically permissive environment in which periodontal breakdown becomes more likely and more severe. Antibiotic-induced shifts in oral and gut microbial communities weaken colonization resistance, support recolonization by hypervirulent taxa, and prime periodontal tissues for exaggerated inflammatory and osteoclastogenic responses. These changes, taken together, identify antibiotic exposure—particularly when recurrent, broad-spectrum, or occurring in early life—as a plausible enhancer of periodontal susceptibility.

Conceptually, antibiotic exposure operates not as a direct aetiologic agent but as a risk-modifying factor that lowers the threshold at which conventional plaque-mediated inflammation progresses to destructive disease. The framing is timely. Global antibiotic consumption is rising, microbial diversity is declining, and antimicrobial resistance is expanding—pressures that converge on the periodontium as on every other host–microbe interface. The hypothesis advanced here is testable: the converging predictions outlined in the Future Directions section, taken together, define a research program that could either substantiate or refute the role of antibiotics as modifiers of periodontal disease susceptibility. In particular, we recommend that this research program be anchored by prospective longitudinal human cohort studies that link well-characterized antibiotic exposure—dose, spectrum, timing, and cumulative burden—to standardized periodontal outcomes, including probing pocket depth, clinical attachment loss, bleeding on probing, and radiographic alveolar bone loss, assessed using consistent case definitions such as the 2017 World Workshop classification and, where feasible, paired with serial oral and gut microbiome profiling. Cohorts of this design, with harmonized measurement protocols and adequate follow-up duration, would supply the causal, human-level evidence that current in vitro and animal data cannot provide. Either outcome would refine antimicrobial stewardship in dentistry, protect microbial resilience, and embed microbiome-preserving principles into periodontal prevention and care.

In terms of clinical implications, the current evidence does not justify any modification of antibiotic prescribing practices specifically on the basis of periodontal risk. Clinicians should continue to prescribe antibiotics according to established, indication-driven stewardship principles, and antibiotics that are genuinely indicated should not be withheld over theoretical periodontal concerns. The practical message of this hypothesis is therefore one of prudent, targeted use and awareness of a possible periodontal dimension to antimicrobial stewardship—not a new, periodontitis-specific prescribing restriction, which the present evidence cannot support.

## Figures and Tables

**Table 1:** Evidence level supporting the principal mechanistic claims linking antibiotic exposure to periodontal susceptibility. For each claim, the predominant level of evidence (in vitro, animal, or human) and its principal translational limitation are indicated, together with the supporting references as cited in the main text.

Mechanistic Claim or Prediction	Section(s)	Evidence Level	Translational Limitation	Ref(s)
Antibiotic depletion of keystone commensals (*S. sanguinis*, *S. gordonii*, *Veillonella*, *Actinomyces*) narrows diversity and opens pathogenic niches	2	Human + animal (microbiome shifts); in vitro (mechanism)	Diversity loss documented in human oral/gut microbiomes; causal link to periodontal breakdown not established	[15,16,17,18]
*P. gingivalis* upregulation of FimA, gingipains, and LPS isoforms under dysbiosis and on rebound	2, 3	In vitro	Molecular/culture systems; not quantified in human post-antibiotic plaque	[19,20,32]
*Candida albicans* β-glucan/dectin-1 amplification of dysbiosis	2	In vitro	Immune-cell and culture models only	[21]
SCFA depletion impairs epithelial barrier and dysregulates NF-κB signalling	2	In vitro + animal (largely gut/IBD)	Extrapolated to oral epithelium; direct oral evidence limited	[22,23,24,25]
Class-specific rebound selection (β-lactam → streptococcal loss; macrolide → FimA; fluoroquinolone → SOS/HGT; tetracycline → *F. alocis*)	3	In vitro/microbiological	Often single-species or high-dose systems	[34,35,36,37]
β-lactam impairment of neutrophil degranulation and killing	4	In vitro	Functional assays; no human gingival crevicular fluid data	[39]
Macrolide suppression of TLR signalling and antimicrobial peptides (LL-37, HBD-2)	4	In vitro/mixed	High or prolonged exposure conditions	[40,41]
Fluoroquinolone NLRP3/IL-1β amplification and RANKL induction	4	In vitro + animal	Concentrations may exceed clinical exposure	[43,44]
Th17 skewing and Treg reduction with tetracyclines/cephalosporins	4	Animal + in vitro	Indirect; not demonstrated in human periodontal tissue	[45,46,47]
Antibiotic osteoblast/osteoclast toxicity (fluoroquinolone apoptosis; aminoglycoside collagen/ALP/Wnt suppression; tetracycline osterix suppression; macrolide osteoclast fusion)	5	In vitro	Frequently supratherapeutic concentrations; clinical relevance uncertain	[51,52,53,54,55,56,57]
Gut dysbiosis → SCFA loss → endotoxaemia → systemic inflammatory priming of the periodontium	6	Animal + human (systemic markers); inferential (periodontal link)	Systemic effects documented; periodontal consequence not directly tested	[60,61,62,63,64,65,66]
Higher cumulative antibiotic use → greater pocket depth/attachment loss	7	No direct human evidence	Epidemiologic literature reviewed in these references does not demonstrate the association; remains hypothesized	[74,75,76,77]

**Table 2:** Summary of evidence linking antibiotic exposure to periodontal susceptibility, organised by mechanistic domain. For each domain, the principal mechanisms, the predominant evidence type (in vitro, animal, or human), and the key knowledge gaps are indicated.

Domain (Section; Figures)	Principal Mechanisms and Current Evidence	Predominant Evidence Type	Key Knowledge Gaps
Oral microbial ecology (Section 2 and Section 3; Figure 1, Figure 2, Figure 3 and Figure 4)	Depletion of keystone commensals narrows diversity and opens niches for opportunistic pathogens (*P. gingivalis*, *T. forsythia*, *F. alocis*); rebound recolonisation may favour more virulent, proteolytic taxa, with class-specific selective effects	Human + animal (diversity shifts); in vitro/microbiological (virulence and rebound mechanisms)	No human data linking post-antibiotic recolonisation to periodontal breakdown; clinical significance of rebound virulence unproven; dose, spectrum, and timing thresholds undefined
Immune regulation (Section 4; Figure 5)	Antibiotic classes alter neutrophil function, TLR signalling, antimicrobial peptides (LL-37, HBD-2), NLRP3 activation, and the Th17/Treg balance, shifting the gingival environment toward a pro-inflammatory, bone-destructive state	In vitro + animal; limited human data	Effects not demonstrated in human gingival or crevicular-fluid samples; exposures often supratherapeutic; reversibility and clinical thresholds unknown
Alveolar bone remodelling (Section 5; Figure 6)	Fluoroquinolones, aminoglycosides, tetracyclines, and macrolides impair osteoblast viability and differentiation and modulate osteoclast activity, increasing alveolar bone vulnerability under inflammatory challenge	In vitro (frequently supratherapeutic doses); some animal	Clinical relevance at therapeutic doses uncertain; no human alveolar-bone data; interaction with inflammatory osteoclastogenesis not quantified in vivo
Gut–oral axis (Section 6; Figure 7)	Gut dysbiosis lowers SCFA production, weakens the intestinal barrier, and promotes endotoxaemia and systemic inflammation (IL-6, TNF-α, IL-1β) that may prime periodontal tissues for exaggerated responses	Animal + human (systemic markers); inferential for the periodontal link	Direct causal connection between gut dysbiosis and periodontal outcomes untested; magnitude of systemic-to-periodontal transfer unknown; confounding by shared risk factors

## Data Availability

The original contributions presented in this study are included in the article. Further inquiries can be directed to the corresponding authors.
